# Environmental effects on survival rates: robust regression, recovery planning and endangered Atlantic salmon

**DOI:** 10.1002/ece3.1614

**Published:** 2015-07-24

**Authors:** Heather D Bowlby, A Jamie F Gibson

**Affiliations:** 1 Bedford Institute of Oceanography, Fisheries and Oceans, CanadaDartmouth, Nova Scotia, B2Y 4A2, Canada; 2 Memorial University of Newfoundland, Ocean Sciences CentreSt. John's, Newfoundland, A1C 5S7, Canada

**Keywords:** Endangered species, freshwater fish, generalized linear models, recovery planning, robust regression, survival analyses, threats

## Abstract

Describing how population-level survival rates are influenced by environmental change becomes necessary during recovery planning to identify threats that should be the focus for future remediation efforts. However, the ways in which data are analyzed have the potential to change our ecological understanding and thus subsequent recommendations for remedial actions to address threats. In regression, distributional assumptions underlying short time series of survival estimates cannot be investigated a priori and data likely contain points that do not follow the general trend (outliers) as well as contain additional variation relative to an assumed distribution (overdispersion). Using juvenile survival data from three endangered Atlantic salmon *Salmo salar* L. populations in response to hydrological variation, four distributions for the response were compared using lognormal and generalized linear models (GLM). The influence of outliers as well as overdispersion was investigated by comparing conclusions from robust regressions with these lognormal models and GLMs. The analyses strongly supported the use of a lognormal distribution for survival estimates (i.e., modeling the instantaneous rate of mortality as the response) and would have led to ambiguity in the identification of significant hydrological predictors as well as low overall confidence in the predicted relationships if only GLMs had been considered. However, using robust regression to evaluate the effect of additional variation and outliers in the data relative to regression assumptions resulted in a better understanding of relationships between hydrological variables and survival that could be used for population-specific recovery planning. This manuscript highlights how a systematic analysis that explicitly considers what monitoring data represent and where variation is likely to come from is required in order to draw meaningful conclusions when analyzing changes in survival relative to environmental variation to aid in recovery planning.

## Introduction

Effective conservation of endangered species and the development of successful recovery plans rely on the identification of environmental and ecological factors limiting population abundance. Small-scale, mechanistic experiments typically reveal environmental parameters that have significant influence on individual characteristics such as growth, habitat use, or physiology (e.g., Nislow et al. 2004; Kiernan and Moyle 2012) and the characteristics that are related to survival rates or population productivity. Subsequently, analyses of an observed time series of abundance data relative to the identified environmental factors are typically used to understand how these mechanisms culminate into changes in survival rates at a population level (Webster 2003; Lawson et al. 2004). However, analyses of temporal trends in data can lack statistical power and give conflicting or nonsignificant results (i.e., type II error) relative to theoretical predictions (Shenk et al. 1998), resulting in the impression that a specific environmental factor is not meaningfully related to population size (and thus should not be the focus of recovery efforts). Therefore, analyses should strive to maximize ecological relevance (in terms of choosing variables for analysis) and to appropriately characterize uncertainty or sources of error to minimize the possibility that significant environmental variation remains undetected (Zuur et al. 2010; Frederiksen et al. 2014). Although this is self-evident for any sound scientific inquiry, how one achieves it when describing species–environment relationships at a population level is equivocal at best (e.g., Hilborn and Walters 1992; Ver Hoef and Boveng 2007).

The validity of conclusions from regression analyses depends in part on appropriately characterizing the distributional form of the response, given that biased estimation resulting from misspecification (i.e., modeling data arising from one distribution with alternate distributions) is well described in the theoretical literature (e.g., Dick 2004). Although survival values should arise from a binomial process: a sequence of Bernoulli trials where an individual is either alive or dead (Collett 2003), the measurement and process errors contributing to estimates of annual abundance also influence the distribution of the relative survival estimates, making it unknown how closely relative survival matches the binomial expectation. In addition, most ecological data exhibits overdispersion relative to the assumed distribution (McCullagh and Nelder 1989). A common way to deal with overdispersion in regression models is to use generalized linear models (GLM) and either the quasilikelihood or the negative binomial family of distributions to estimate the variance (e.g., Ver Hoef and Boveng 2007). However, this only accounts for atypical values in the response. An alternative would be to use a regression method that estimates the functional relationship between the predictors and response in situations where the underlying assumptions are violated to some extent (i.e., either the predictor or response contains atypical values). Termed robust regression (e.g., Hampel et al. 1986; Heritier et al. 2009), these methods offer several distinct advantages over more commonly used regression techniques, including an increased ability to detect a subtle signal in noisy data as well as the ability to produce unbiased estimates of variance around a fitted relationship for overdispersed data (Cantoni and Ronchetti 2001). Starting from a parametric model (i.e., a particular model form as in a GLM), robust regression builds in protection against outlying behavior in the data during the estimation process, by reducing the influence of atypical values on the objective function (Hampel et al. 1986). As such, the robust counterpart to a GLM should not be considered a competing model per se, but rather a method by which to (1) identify atypical values or outliers in a dataset (relative to what is assumed a priori by the model) and (2) to reduce bias in the estimated coefficients (particularly the variance) which result from these values.

The primary goal of this study was to quantitate changes in survival relative to environmental variation for use in recovery planning. In doing so, we explored the implications of common assumptions and methods when attempting to describe environmental relationships and demonstrated how our understanding partially depends on the statistical technique and assumed distributional form of the response chosen prior to the analytical process. Although the conclusions were framed relative to a specific application, the methods are directly applicable to recovery planning for multiple species in which observational time series of abundances are available to estimate survival rates. Using a case study of juvenile Atlantic salmon *Salmo salar* (Linnaeus, 1758) survival relative to variation in hydrological flow, we demonstrated how restricting the analyses to traditional regression techniques would have led to the identification of multiple significant hydrological predictors, yet low overall confidence in the predicted relationships. However, using robust regression to evaluate the effect of additional variation and outliers in the data relative to regression assumptions resulted in a better understanding of the relationships between hydrological variables and survival that could be used for population-specific recovery planning.

## Case study

For endangered Atlantic salmon populations, there is considerable interest from multiple nongovernment organizations, academics, and government departments to implement remedial actions at a watershed scale to promote population increase. Many of the actions related to habitat enhancement (e.g., bank stabilization, digger logs, changing channel morphology) are proposed because of their influence on hydrological flows, with the assumption being that such changes will increase the productive capacity of freshwater environments for Atlantic salmon (Roni et al. 2002). Hydrological variation is thought to be a key factor controlling the population dynamics of freshwater fishes, in that it influences the majority of physical factors (e.g., current velocity, water depth, and temperature regime) and ecological interactions (e.g., competition, predation) experienced by fish in freshwater environments (Bunn and Arthington 2002; Kiernan and Moyle 2012). Five major components of flow are considered to be ecologically important across a diverse range of riverine ecosystems: extreme low flows, low flows, high flow pulses, small floods, and large floods (Mathews and Richter 2007; Poff et al. 2010). Under this categorization, low flows represent typical flow conditions which determine the amount and characteristics (e.g., temperature, connectivity, and velocity) of aquatic habitat available for the majority of the year. The other flow categories are thought of as discrete events that typically trigger a behavioral response (Mathews and Richter 2007) and thus might be correlated with survival rates. Extreme low flows describe drought conditions, which are characterized by a decrease in surface area and water volume causing extreme values of several physical and chemical water quality parameters, such as temperature, flow velocity, oxygen concentration, or dissolved mineral content (Magoulick and Kobza 2003; Rolls et al. 2012). For aquatic species, droughts induce stress responses and typically increase mortality due to a reduction in habitat connectivity, availability, and suitability (Lake 2003). Thus, it might be expected that increased frequency or severity of drought conditions experienced by juvenile Atlantic salmon would result in measureable declines in survival rates at a population level. Conversely, high flow pulses (up to bankfull) are thought to recharge river systems by reducing water temperatures, flushing wastes, increasing oxygen availability, and delivering organic matter (Mathews and Richter 2007) and thus would be expected to be positively correlated with survival. However, large floods or quick changes in water level are considered to be less directly beneficial for individuals given that they can move significant amounts of sediment and large woody debris, transport organisms downstream, and alter the direction of the main channel. However, in the long term, they also form new habitats and refresh water quality conditions in stagnant portions of the stream (Allan 2004). Given that a decline in survival related to flood conditions is predicted to come from sediment transport and displacement (Caissie 2006), the rise rate of the river could have a more direct influence on survival than flood conditions per se. Understanding how low or high water conditions influence juvenile survival in specific populations would be a first step toward identifying whether or not hydrological change should be a focus of recovery efforts, as well as which specific components of the flow regime should be targeted in specific watersheds.

### Data sources

**Figure 1:**
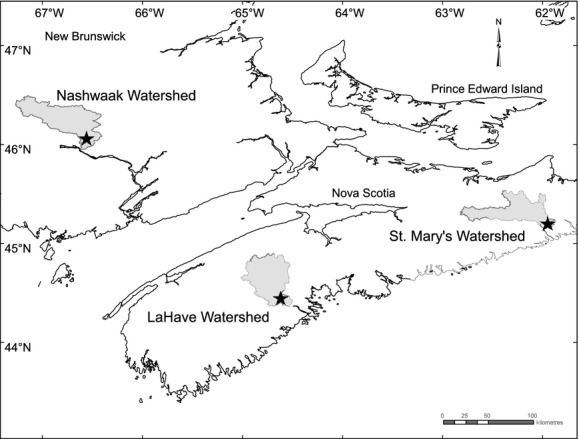
Location of the study area in Atlantic Canada showing the boundaries of the St. Mary's, LaHave and Nashwaak watersheds as well as the locations of the hydrological monitoring stations (stars).

Population monitoring data collected from the west branch of the St. Mary's River, the LaHave River above Morgans Falls (both in Nova Scotia, Canada) as well the Nashwaak River in New Brunswick, Canada (Fig. 1), were used in this study. Of the Atlantic salmon populations considered to be endangered by the Committee on the Status of Endangered Wildlife in Canada (COSEWIC 2010), these rivers are the only three in the Maritime Provinces that (1) have long-term monitoring programmes which enumerate all freshwater life stages for at least a portion of the watershed and (2) have hydrological monitoring stations gauging daily water flows in a location near to that for the population monitoring data. The annual egg deposition and juvenile density estimates used for analyses came from recent assessments (Gibson and Bowlby 2012 [St. Mary's and LaHave]; and Gibson et al. In press [Nashwaak]) in which annual egg depositions were estimated from the number and characteristics of adult spawners, and age 0 densities were estimated from electrofishing surveys. Age 0 salmon were those sampled in the year of hatching as juveniles between June and September. A Poisson GLM (incorporating site and year effects) was used to predict age 0 density values for all potential sites (from a random-stratified survey design) prior to calculating the annual mean densities. This standardization was performed to reduce annual estimation error and account for any directional biases related to changes in site selection (Gibson et al. 2009). For these populations, it has been shown to produce density estimates that are more consistent with data available for other life stages when analyzed in age- and stage-structured population dynamics models (e.g., Gibson and Bowlby 2012). All data were analyzed relative to a specific egg cohort, with age 0 density lagged by 1 year relative to egg deposition for calculating survival. Egg deposition estimates were scaled by area to make them comparable to age 0 density estimates. Although compensatory density dependence (Rose et al. 2001) would attenuate any density-independent effect of environmental variation on survival, previous research (Gibson 2006) found very little evidence of density dependence from the time of egg deposition to the time of electrofishing in the following year (i.e., egg to age 0), but comparatively strong evidence for density dependence from age 0 to age 1.

Environment of Canada maintains hydrological gauging stations on the St. Mary's River at Stillwater (45°10′27″N 61°58′47″W), on the LaHave River at West Northfield (44°26′50″N 64°35′28″W), and on the Nashwaak River at Durham Bridge (46°07′33″N 66°36′40″W) (Fig. 1). These stations have been in operation continuously from 1915 on the St. Mary's and LaHave Rivers and 1961 on the Nashwaak; historical flow data can be downloaded from the Water Survey of Canada's HYDAT database of archived hydrometric data (http://www.ec.gc.ca/rhc-wsc/default.asp?lang=En&n=9018B5EC-1, Accessed May 2013). Daily values (in cubic meters per second) correspond to averages of hourly recordings, and linear extrapolation was used to estimate values for days during which the station was not operational (1.6%, 0.7% and 1.8% of the time series for the St. Mary's, LaHave and Nashwaak Rivers, respectively; considering all years up to 2010).

### Survival-hydrology analyses

Hydrological variables describing flow conditions were calculated from the environmental flow components module of the indicators of Hydrologic Alteration software (Mathews and Richter 2007). This module categorizes daily flows into the five ecologically important components identified earlier (extreme low flows, low flows, high flows, small floods, and large floods) based on user-defined thresholds. From these categories, four annual variables were calculated: (1) the minimum flow value (i.e., the lowest flow value recorded), (2) the frequency of extreme low flows (i.e., the number of days categorized as having extreme low flows), (3) the timing of extreme low flows (i.e., the median ordinal date of all the days classified as extreme lows), and (4) the rise rate (i.e., the median of all positive differences between two daily flow measurements). This value represents how quickly water levels increase following precipitation events or snowmelt and does not depend on the initial water conditions in the river (i.e., the classification of each flow measurement as extreme low, low, or high flows; small or large floods). The lowest 20% percentile (of all flow measurements regardless of year) was used as the cutoff between extreme low flows and low flows to ensure that a value could be calculated for all parameters for all years. For high water conditions, all flows categorized as high flows, small floods and large floods would have had to be combined in order to calculate values for all parameters for all years, even though these would be expected to have opposing relationships with survival. Instead, the rise rate was used as an indicator of the flashiness of the river system and the potential for bedload transport (Caissie 2006), with the expectation that faster rise rates would negatively affect survival. Based on Kendall's *tau*, correlations among predictor variables were <0.6. To ensure that the hydrological conditions corresponded to the time period between autumn egg deposition and juvenile sampling the subsequent summer, a year was considered to begin on November 1 and end at the start date of the summer electrofishing survey (July to September).

Survival or mortality can be thought of multiple ways, leading to different response variables and model structures for regression analyses. Here, hydrological relationships with the estimated density of age 0 juveniles could be modeled directly, assuming a Poisson distribution for age 0 abundance (appropriate for count data), a log link, and including an offset for starting population size (egg deposition) in a GLM (McCullagh and Nelder 1989). A second alternative would be to calculate a survival rate (age 0 density divided by the previous year's egg deposition), which could be modeled as a binomial process with a logit link using a GLM (McCullagh and Nelder 1989). The third option would be to model the instantaneous mortality rate assuming a normal error distribution as in a linear regression. Survival is related to mortality by *S* = *e*^−*Zt*^ so the instantaneous mortality rate (*Z*) is: *Z* = −ln (*S*) (Ricker 1975). Fitting a linear regression model to a log-transformed response (i.e., the instantaneous mortality rate) is equivalent to fitting a multiplicative model with lognormal errors (Dick 2004). It is important to note that the lognormal model would be expected to have a slope estimate opposite in sign as compared to the other regressions. Starting from these three models (count data with Poisson errors, a survival rate with binomial errors, and the instantaneous mortality rate with lognormal errors), we used two different methods to account for potential overdispersion in the GLMs. One was to substitute the quasibinomial and quasi-Poisson family into the GLMs described above, which estimates a dispersion parameter for the variance. The second was to assume a negative binomial distribution when modeling age 0 density (with an offset for the previous year's egg deposition) in a GLM (Ver Hoef and Boveng 2007). The results in this manuscript are presented for the lognormal, quasibinomial, quasi-Poisson, and negative binomial models, as detailed in Table 1. For the 2 years in which estimated survival was >1 on the St. Mary's River, survival was set at 1 in order to be able to fit the quasibinomial, quasi-Poisson, and negative binomial models.

**Table 1:** Description of the lognormal and generalized linear model (GLM) forms considered for analyzing egg to age 0 survival data for Atlantic salmon from three populations, detailing the response variable, response distribution, parametric model, and variance estimator. Terms used are as follows: hydrological predictors (*X*_*n,i*_), mean value (*μ*_*i*_), probability of being alive (*π*_*i*_), age 0 density (*λ*_*i*_), egg density (*n*_*i*_), 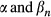
are the regression coefficients, *θ* is an overdispersion parameter, and *κ* is the scale parameter from a gamma distribution

Model	Dependent variable	Response distribution	Parametric model	Variance
Lognormal	−ln (*S*)	*Y*_*i*_ ∼ *N*(*μ*_*i*_, *σ*_*i*_)	*α* + *β*_1_*X*_1,*i*_ + …*β*_*n*_*X*_*n*,*i*_	*σ*^2^
Quasibinomial (with offset)	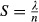	*Y*_*i*_ ∼ *B*(*n*_*i*_, *π*_*i*_)	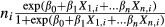	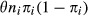
Quasi-Poisson (with offset)	*λ*	*Y*_*i*_ ∼ *P*(*μ*_*i*_)	ln (*λ*_*i*_) - ln (*n*_*i*_) = *β*_0_ + *β*_1_*X*_1,*i*_ + …*β*_*n*_*X*_*n*,*i*_	*θμ*_*i*_
Negative binomial (with offset)	*λ*	*Y*_*i*_ ∼ *NB*(*μ*_*i*_, *κ*)	ln (*λ*_*i*_) - ln (*n*_*i*_) = *β*_0_ + *β*_1_*X*_1,*i*_ + …*β*_*n*_*X*_*n*,*i*_	*μ*_*i*_(1 - *μ*_*i*_*κ*)

Atypical values (outliers) and points with high leverage are known to bias parameter estimation using maximum likelihood as in GLMs (Graham 2003). Additionally, the specific data points contributing to such biases as well as the magnitude and direction of the bias cannot be assessed statistically from the output of GLM regressions (Richards 2008; Zuur et al. 2010), although several ad hoc methods of identifying outliers exist (e.g., visual examination of residual plots). Robust regression provides a statistical framework from which to both identify and limit the influence of extreme values or leverage points on parameter estimation. Depending on the specific method used, up to half of the data can take atypical values and still have limited influence on coefficient estimates (e.g., Hampel et al. 1986; Yohai 1987). Therefore, we used robust regression more as an extension of the traditional linear and GLMs, both to evaluate the presence of outliers and to obtain less biased estimates of regression coefficients for use in recovery planning.

Robust regression uses Mallows- or Huber-type robust estimators (typically called M-estimators; Jajo 2005; Cantoni and Ronchetti 2001) to estimate model parameters. Although the postulated model (i.e., the assumed distribution of the response and associated linear predictor) used in robust analyses is analogous to that used in traditional regressions or GLMs (Table 1), estimation of the *β* parameters proceeds in a different manner. As a simple example, it is useful to compare the familiar least-squares estimator with an M-estimator to appreciate the main differences among the two techniques. For a linear model, the least-squares estimator for *β* minimizes the objective function: 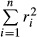, where each residual (*r*_*i*_) is: 
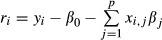
 for each value of the response variable, *y*_*i*_, and each value of *p* hydrological predictors (*x*_*i,j*_). An M-estimate of *β* minimizes the objective function: 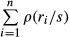, where *s* is an estimated scale parameter and *ρ* is called the psi function. Note that if the weighting function *ρ*(*r*_*i*_/*s*) is equivalent to *r*_*i*_^*2*^, the parameter estimates will be the same as from ordinary least squares (Jajo 2005). The influence of individual residuals on model fitting is controlled by the derivative of the psi function: *ψ* Multiple functions can be chosen for *ψ*, but each has the common characteristic of limiting the contribution of data points that deviate substantially from the fitted relationship. The scale parameter can be thought of as a multiplier on the error term, representing deviation from the assumed error distribution. A simple M-estimate (as above) was not appropriate for this application given that the levels of the predictor were not fixed a priori (Maronna et al. 2006). Here, we used MM-estimation in the lmrob function for fitting a robust lognormal model (Yohai 1987) and the Mqle method in the glmrob function for fitting robust binomial and robust Poisson models (Cantoni and Ronchetti 2001), as implemented in the readily available R package “robust base” (Rousseeuw et al. 2013). We followed recommendations for the tuning constants from Koller and Machler (2013) for the robust lognormal and robust binomial models (*k* = 4.685 for the redescending *ψ* used in lmrob; *k* = 1.345 for the Huber *ψ* in glmrob). We increased the tuning constant used for the robust Poisson model slightly (*k* = 1.8) for both rivers. Given that there are no robust counterparts to the quasifamily GLMs, we employed the newly available “glmrob.nb” function (Aeberhard et al. 2014) to allow for overdispersion in the response for a robust model. Here, we used the redescending Tukey's biweight function for *ψ* in the M-estimates of the regression parameters and the same tuning constant as above (e.g., *k* = 0.4685).

In total, we evaluated 4 potential hydrological predictors using the 4 parametric models (Table 1) as well as the two different regression types (robust and traditional) and presented the results from eight models (lognormal, quasibinomial, quasi-Poisson, negative binomial, robust lognormal, robust binomial, robust Poisson, and robust negative binomial). Model selection proceeded in three general steps: (1) simplification of the initial multivariate model using traditional linear regression or GLMs, (2) evaluation of regression assumptions from diagnostic plots (all traditional models) and estimated overdispersion parameters (quasifamily models), and (3) evaluation of the effect of atypical values on the predicted coefficients using robust regression. Hydrological predictors were both sequentially added and dropped from each regression based on the significance of individual terms in the fitted model (*P*-value) as well as a comparison of nested models using ANOVA (for lognormal models), Likelihood ratios (for GLM models; Zuur et al. 2009), and the Robust Wald test (for robust lognormal or robust GLMs; Sommer and Huggins 1996). In all regressions, the final model included a single predictor. Diagnostic plots of the residuals, quantiles and fitted versus observed values were examined visually for each model to assess the appropriateness of model assumptions (Zuur et al. 2009, 2010). If significant autocorrelation was detected in the residuals, we used AIC to compare the fit from a generalized least squares (GLS) model with a residual first-order correlation structure (ar1) to the GLM fit (Zuur et al. 2009) and re-evaluated the significance of the hydrological predictors. For the results presented, model diagnostics were similar (i.e., there was no compelling reason to reject individual models based on diagnostic plots), so the assumptions underlying each model appeared to be appropriate.

Traditionally, model selection for regression analyses uses an information theoretical approach such as the Akaike information criterion (AIC) or the Bayesian information criterion (BIC); both of which assess fit from maximum-likelihood scores that are penalized for model complexity (Johnson and Omland 2004). This presents a problem when attempting to compare among the GLMs presented here (i.e., to compare the best-supported models for each distributional form of the response after variable reduction) because the quasifamily is characterized by a mean and variance but not a specified distributional form, which means that the log-likelihood is not defined (Ver Hoef and Boveng 2007). Therefore, it is not possible to use a statistical criterion such as AIC to evaluate model fits from all 4 traditional regressions (lognormal, quasibinomial, quasi-Poisson, and negative binomial). However, the lognormal and negative binomial models could be directly compared with the Akaike information criterion for small samples (AICc), and the quasifamily models could be compared using a quasi-AIC for small samples (QAICc) (e.g., Young et al. 2009). Further to this, it is possible to assess the appropriateness of the quasifamily models via the variance inflation factor (model deviance divided by residual degrees of freedom) (Collett 2003), where values are expected to be less than ∼4 when the data structure is well specified (Anderson et al. 1994). In relation to the robust models, the estimated coefficients would be essentially identical to those estimated from traditional regressions if model assumptions were true (i.e., provided that variation in the response conformed exactly to the assumed distribution and the predictors did not contain atypical values). However, as compared to GLMs assuming the same distribution of the response, robust regressions have greater statistical efficiency (reduced variance) and can produce unbiased estimates of coefficients if assumptions are violated to some extent (Hampel et al. 1986; Jajo 2005; Heritier et al. 2009). Therefore, we considered the robust regressions to be the way in which we could reduce the potential for type II error and obtain better estimates of coefficients for use in recovery planning, relative to the equivalent traditional model (*c.f*. lognormal with robust lognormal, binomial with robust binomial, Poisson with robust Poisson, and negative binomial with robust negative binomial).

## Results

**Figure 2:**
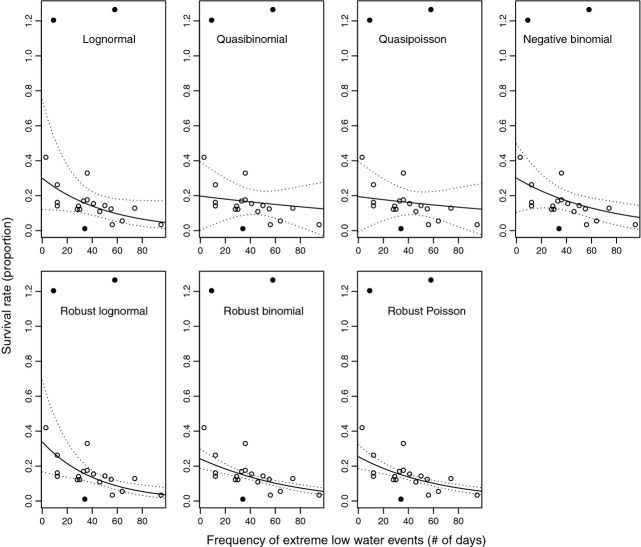
A comparison of the fits of seven different regression models to egg to age 0 survival data (egg cohorts: 1989–2009) relative to the frequency of extreme low water events from the St. Mary's River, showing the observed values (points), the predicted fit of the model (lines) and 2*SE (dashed lines). The response variable was standardized to be an annual survival rate to facilitate comparison. Although the preferred quasi-Poisson model retained an alternate predictor as significant (Table 2) and the quasibinomial model retained no significant predictors, the nonsignificant relationship with the frequency of extreme low flows is shown here. To date, a predict function has not been developed for the newly available robust negative binomial model, which is why the results are not included here (although see Table 2).

For the St. Mary's River, the lognormal and GLM regressions did not consistently simplify to the same hydrological predictor. Survival was found to be negatively associated with the frequency of extreme low water events (xlow.freq) from the lognormal and negative binomial models, while the quasi-Poisson model identified a positive relationship with the timing of extreme low water events (dist.low) and the quasibinomial model revealed no significant predictors (Table 2). The lognormal model had a significantly better fit than the negative binomial (AICc = 64 and 147, respectively) using xlow.freq as the predictor. For the quasi-Poisson model of survival relative to dist.low, the estimated overdispersion parameter (12.05) was substantially >4, indicating that this model was not an adequate characterization of the data (Anderson et al. 1994), even though the predictor was retained as significant. Recalculating the response to be a survival rate and comparing the fits of the 4 traditional regressions with xlow.freq revealed only minor deviations in the predicted mean slope (Fig. 2). As above, the estimated overdispersion parameters for the quasi-Poisson and quasibinomial models of survival relative to xlow.freq were unacceptably high (16.16 and 12.05, respectively). Overall, the lognormal model was considered to be the best model structure with which to describe the relationship between hydrological change and survival for the St. Mary's River. When the data were re-examined in the robust analyses, all four robust models found survival to be negatively associated with the frequency of extreme low water events (Table 2). This suggests that the weak relationship predicted between survival and dist.low from the quasi-Poisson GLM was spurious and caused by outliers or points with high leverage in the data. The lognormal and robust lognormal models had identical slope estimates (*c.f*. 0.064 and 0.064; Table 2), indicating that atypical values had no influence on this parameter estimate. However, the 95% confidence intervals (based on the normal approximation) are much smaller for the robust model, particularly at lower survival values (e.g., compare the lognormal and robust lognormal fits; Fig. 2). The robust lognormal model identified three of 21 data points that were contributing substantially to this difference, in that they were given a weighting (a robustness weighting that corresponds to the *ψ* function of the residual divided by the residual) of <0.7.

**Table 2:** Comparisons of coefficients from eight regression model forms describing egg to age 0 survival relative to the frequency of extreme low water conditions (xlow.freq) for the St. Mary's River and the median rise rate (rise.rate) for the Nashwaak River. Coefficients from a model that retained an alternate hydrological predictor (the timing of extreme low water events; dist.low) for the St. Mary's River are also shown. Note that the slope estimates for the models of mortality rates would be expected to be opposite in sign to those of survival rates or age 0 density. Results from the LaHave River are not included because no significant predictors were identified

River	Model	Dependent variable	Independent variable	Value	SD	*P*-value
St. Mary's	Lognormal	Instantaneous mortality rate	xlow.freq	0.064	0.027	0.030
St. Mary's	Quasibinomial	Annual survival rate	xlow.freq	−0.046	0.038	0.240
St. Mary's	Quasi-Poisson	Age 0 density (offset pop size)	xlow.freq	−0.039	0.031	0.235
St. Mary's	Negative binomial	Age 0 density (offset pop size)	xlow.freq	−0.054	0.021	0.010
St. Mary's	Robust lognormal	Instantaneous mortality rate	xlow.freq	0.064	0.020	0.005
St. Mary's	Robust binomial	Annual survival rate	xlow.freq	−0.055	0.007	≪0.001
St. Mary's	Robust Poisson	Age 0 density (offset pop size)	xlow.freq	−0.048	0.003	≪0.001
St. Mary's	Robust negative binomial	Age 0 density (offset pop size)	xlow.freq	−0.059	0.007	≪0.001
St. Mary's	Quasi-Poisson	Age 0 density (offset pop size)	dist.low	0.014	0.006	0.038
Nashwaak	Lognormal	Instantaneous mortality rate	rise.rate	0.326	0.114	0.007
Nashwaak	Quasibinomial	Annual survival rate	rise.rate	−0.299	0.138	0.037
Nashwaak	Quasi-Poisson	Age 0 density (offset pop size)	rise.rate	−0.247	0.119	0.045
Nashwaak	Negative binomial	Age 0 density (offset pop size)	rise.rate	−0.256	0.108	0.018
Nashwaak	Robust lognormal	Instantaneous mortality rate	rise.rate	0.401	0.137	0.006
Nashwaak	Robust binomial	Annual survival rate	rise.rate	−0.420	0.052	≪0.001
Nashwaak	Robust Poisson	Age 0 density (offset pop size)	rise.rate	−0.294	0.046	≪0.001
Nashwaak	Robust negative binomial	Age 0 density (offset pop size)	rise.rate	−0.460	0.109	≪0.001

**Figure 3:**
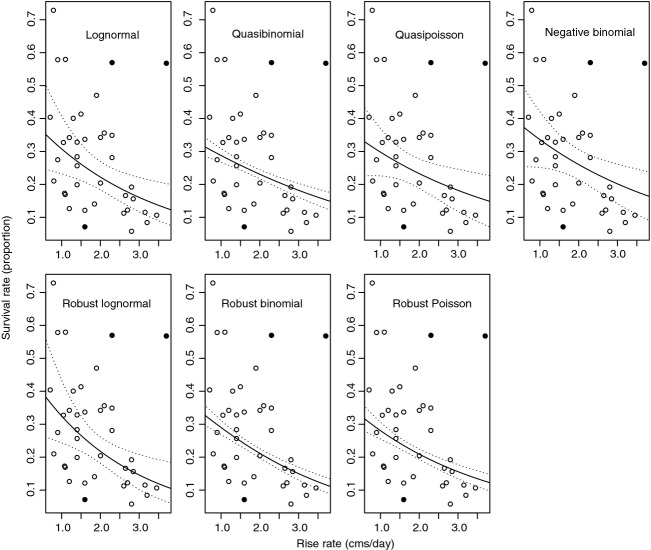
A comparison of the fits of seven different regression models to egg to age 0 survival data (egg cohorts: 1970–2009) relative to the rise rate (cms/day) from the Nashwaak River, showing the observed values (points), the predicted model fit (lines), and 2*SE (dashed lines). The response variable was standardized to be an annual survival rate to facilitate comparison. To date, a predict function has not been developed for the newly available robust negative binomial model, which is why the results are not included here (although see Table 2).

For the Nashwaak River, the different model forms were more consistent in that they all identified a negative relationship between egg to age 0 survival and the rise rate of the river (Table 2). Slope estimates from the quasibinomial, quasi-Poisson, and negative binomial GLMs were very similar (−0.299, −0.247, and −0.256, respectively; Table 2), although there was minimal support for the two quasifamily models based on their *P*-values. As was the case for the St. Mary's River, the estimated overdispersion parameters for the quasibinomial and quasi-Poisson models were quite high (8.10 and 7.68, respectively), again indicating that these models do not adequately describe the data. Based on AICc, the lognormal model gave a significantly better fit to the data as compared to the negative binomial GLM (AICc = 74 and AICc = 298, respectively) and was considered to be the best model structure with which to describe these data. Similar to the St. Mary's, the robust weightings indicate that the data are approximately lognormal, given that only three of 39 points are downweighted by more than 0.7 (Cantoni and Ronchetti 2001). However, these outliers have a greater influence on the predicted coefficients in that the robust lognormal model predicted a greater decline in survival (i.e., more negative slope) as rise rate increased relative to the lognormal model (Table 2; Fig. 3).

For the LaHave River, initial fits from the lognormal and GLM models found survival to be related to the frequency of extreme low water events (xlow.freq), but had strongly autocorrelated residuals at a lag of 1. Re-analysis in a generalized least squares model with an ar1 residual correlation structure significantly reduced model AIC, but xlow.freq was no longer a significant predictor in the model (*P*-value = 0.07). Therefore, a relationship between egg to age 0 survival and hydrological change could not be described for the LaHave River population from these analyses.

## Discussion

Investigating changes in survival relative to environmental variation requires a systematic analysis that explicitly considers what the monitoring data represent and where variation is likely to come from in order to draw meaningful conclusions. It is particularly important in cases where the distributional assumptions underlying the methods cannot be investigated a priori (Ver Hoef and Boveng 2007; Zuur et al. 2010), as well as in situations where both predictors and responses could contain variation that is unaccounted for with a particular model structure (Richards 2008). The relative popularity of GLMs stems from their ability to account for alternate mean–variance relationships and errors arising from certain types of biological processes (McCullagh and Nelder 1989). At first glance, GLMs may have been expected to be the most appropriate method for analyzing juvenile salmon survival relative to hydrological variation given that the observational time series derive from count data and survival is an inherently binomial process. However, our analyses suggest that the appropriate error distribution for the survival estimates deviates from theoretical expectations, likely due to the combined observation and measurement error associated with the population monitoring data.

Simulation studies have demonstrated that violating regression assumptions can produce spurious correlations or can mask significant correlations when data contain additional errors in the predictors or response (Graham 2003). Both of these potential biases were demonstrated by the GLM models for the St. Mary's River, with the quasi-Poisson model showing a seemingly spurious correlation with the timing of extreme low flows, and the relationship between survival and the frequency of extreme low water events being masked in the quasifamily models. The most obvious outliers (i.e., the survival estimates >1) occurred when the median timing of extreme low flows was later in the year (i.e., at higher values of dist.low) and would be expected to have high leverage on model fits. It is likely that dist.low was only retained as a significant predictor by the quasi-Poisson model because of characteristics of the estimation process. For example, quasi-Poisson regression gives greater weight to larger counts in the fit from iteratively weighted least squares as compared to alternatives such as the negative binomial (Ver Hoef and Boveng 2007), and the age 0 densities contributing to the survival estimates above one were an order of magnitude larger than the majority of the other values. Although survival values greater than one could have been removed because they were not biologically plausible, this would have been the equivalent of preferentially excluding data points when survival would be expected to be high. As an alternative, robust methods are a powerful way to analyze data that is subject to measurement and process error in that they do not require any a priori assessment of data quality (i.e., removal of biologically implausible values or other outliers). Because of the downweighting imposed by the influence function during estimation, the survival values greater than one would have little influence on model fit. Therefore, the robust regressions should have also identified the relationship between survival and the timing of low water events (dist.low) if it was unrelated to leverage points in the data. Similarly, the impact of outliers was found to be relatively small on the slope estimates for the St. Mary's River (*c.f*. traditional and robust parameter estimates; Table 2), but larger on the standard deviation. This influences the significance of parameters in the model and is likely why the quasibinomial and quasi-Poisson GLMs did not retain xlow.freq as a predictor.

The practical consequences of such statistical considerations can be quite large for this type of a research question. Restricting these analyses to GLMs (e.g., the quasibinomial, quasi-Poisson and negative binomial models) would have led to ambiguous results among candidate models for the St. Mary's River as well as to slight confidence in the predicted relationships on the Nashwaak River. Furthermore, it would not be immediately obvious whether the assumed distribution was inappropriate, the underlying relationships were weak (i.e., not ecologically important), or if variability in the data (i.e., violations of assumptions) was adversely affecting parameter estimation. Taking this one step further for recovery planning, the GLMs would not form as convincing a basis to argue that remediation actions to alter hydrological flows should be included in a remediation strategy. Extending the analyses using both the lognormal model and robust regression enabled us to address all of these uncertainties and to identify the hydrological predictor best supported by the data (xlow.freq on the St. Mary's River and rise.rate on the Nashwaak), as well as to reduce biases in the estimated coefficients. The latter was particularly important on the Nashwaak River, given that the estimated slope increased from approximately 0.3–0.4, indicating closer to a fourfold rather than a threefold change in the instantaneous mortality rate over the range of observed rise rates (Fig. 3).

The identification of population-level changes in survival to hydrological variation gives indirect evidence for the specific threats that have resulted in population decline in these three rivers as well as the expected population response to recovery actions. Furthermore, it would be expected that changes in hydrological conditions that have resulted in increased contrast in the data (i.e., anthropogenic activities that cause more extreme flow values) would enhance our ability to detect relationships with flow (Frederiksen et al. 2014). The primary anthropogenic activities that have been linked to changes in hydrological flow patterns are related to land clearing (Allan 2004; Broadmeadow and Nisbet 2004; Poff et al. 2006), which can result from mining operations, urbanization, agriculture, or forestry, and the effects of which can be exacerbated by changing precipitation patterns due to climate change (Milly et al. 2005). Extreme low water conditions can arise from a reduced capacity of the watershed to retain runoff owing to the removal of vegetation (Broadmeadow and Nisbet 2004) as well as to water extraction from surface water or aquifers (Allan 2004). Recovery plans that identify the specific location, extent and severity of such activities, and remedial actions designed to alleviate these threats would be expected to have a positive influence on egg to age 0 survival on the St. Mary's River. The speed at which water levels increase is related to geology and vegetation patterns which determine the capacity of a drainage area to absorb runoff (Jewett et al. 1995; Allan 2004), as well as to channel morphology, where straighter, deeper streambeds enable faster water flow (Paul and Meyer 2001). Given that approximately 90% of the Nashwaak River watershed was clear-cut in 1978–1979 (Jewett et al. 1995), our ability to detect the negative relationship between egg to age 0 survival and hydrological rise rate may represent the effect of a land-use legacy (Greenwood et al. 2012), by increasing the contrast in the data for this Atlantic salmon population. Remediation focused on riparian planting, minimizing erosion, and sources of sedimentation, as well as increasing channel complexity, would be expected to result in increased egg to age 0 survival in the Nashwaak River. For the LaHave River, autocorrelation in the residuals was the strongest signal found in the data, indicating a decline in egg to age 0 survival over the duration of monitoring that was not related to hydrology. These results do not preclude the possibility that a relationship between egg to age 0 survival and alternate hydrological predictors exist, nor that additional data collection and a longer time series would enable a relatively weak relationship to be described. However, in terms of guiding recovery planning, alternate threats that are not as strongly linked to hydrology, such as the effects of invasive smallmouth bass and chain pickerel (Wathen et al. 2011) or changes to water quality (Paul and Meyer 2001) should be investigated.

This manuscript provides one example of how our interpretation of ecological data changes as a result of the assumptions made during the analytical process and highlights the implications that these assumptions can have for future recovery planning. Given the declining trends in a large number of freshwater fish species (e.g., Dudgeon et al. 2006) as well as the limited time and resources available for remediation, the efficient identification of priorities for recovery planning is a pressing ecological issue.
